# Kirigami‐Inspired Programmable Soft Magnetoresponsive Actuators with Versatile Morphing Modes

**DOI:** 10.1002/advs.202203711

**Published:** 2022-09-30

**Authors:** Hanlin Zhu, Yuan Wang, Yangwen Ge, Yan Zhao, Chao Jiang

**Affiliations:** ^1^ State Key Laboratory of Advanced Design and Manufacturing for Vehicle Body College of Mechanical and Vehicle Engineering Hunan University Changsha 410082 P. R. China

**Keywords:** bionic soft crawling robots, gaussian curvature, kirigami‐inspired design, mechanical assembly, programmable soft magnetoresponsive actuators

## Abstract

Untethered soft magnetoresponsive actuators (SMRAs), which can realize rapid shape transformation, have attracted widespread attention for their strategic applications in exploration, transportation, and minimally invasive medicine. It remains a challenge to fabricate SMRAs with complicated morphing modes (more than bending and folding), limiting their applications to simple shape‐morphing and locomotion. Herein, a method integrating the ancient kirigami art and an advanced mechanical assembly method is proposed, which realizes 2D‐to‐3D and 3D‐to‐3D complicated shape‐morphing and precise magnetization programming through cut‐guided deformation. The kirigami‐inspired SMRAs exhibit good robustness after actuating more than 10000 times. An integrated finite element analysis method is developed to quantitatively predict the shape transformation of SMRAs under magnetic actuation. By leveraging this method, a set of 3D curved responsive morphologies with programmed Gaussian curvature are fabricated (e.g., ellipsoid and saddle structures), specifically 3D multilayer structures and face‐like shapes with different expressions, which are difficult to realize using previous approaches. Furthermore, a bionic‐scaled soft crawling robot with significant obstacle surmounting ability is fabricated using the kirigami‐inspired method. The ability of the method to achieve programmable SMRAs with versatile morphing modes may broaden its applications in soft robotics, color‐switchable devices, and clinical treatments.

## Graphical abstract

Kirigami‐inspired programmable soft magnetoresponsive actuators (SMRAs) are experimentally, theoretically, and numerically investigated. Three‐dimensional (3D) complicated and direction‐varying magnetization profiles can be obtained by cut‐guided deformation and magnetization programming. The SMRAs can transform into 3D curved morphologies with complicated Gaussian curvatures. Diverse functional applications, including various 3D face shapes and a bionic‐scaled soft crawling robot, are demonstrated.

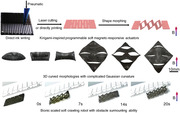

## 1 Introduction

Soft actuators have attracted wide and increasing interest because of their great potential in deep‐water exploration,^[^ ^1^, ^2^ ^]^ object transportations,^[^ ^3^, ^4^, ^5^, ^6^, ^7^ ^]^ and bioengineering.^[^ ^8^, ^9^, ^10^ ^]^ In addition, soft actuators with programmable shape‐morphing abilities are highly desired for realizing object manipulation in complicated and limited environments.^[^ ^11^ ^]^ Stimuli‐responsive soft materials are widely used to fabricate soft actuators because they can realize various shape deformations or functional operations driven by various stimuli, such as light,^[^ ^12^, ^13^ ^]^ heat,^[^ ^14^ ^]^ electric fields,^[^ ^15^ ^]^ and magnetic fields.^[^ ^8^, ^16^, ^17^ ^]^ Specifically, magnetoresponsive soft materials that can realize rapid, reversible, and programmed shape morphing under remote and untethered actuation are particularly desirable for the preparation of soft machines with excellent environmental adaptability.^[^ ^6^, ^18^, ^19^, ^20^, ^21^, ^22^, ^23^, ^24^, ^25^, ^26^ ^]^

Soft magnetoresponsive actuators (SMRAs) are driven by the magnetic moment generated from the magnetic fillers embedded into the polymer matrix under magnetic actuation. The magnetic moment aligns the magnetization direction of all magnetic domains with the applied magnetic field direction, leading to macrodeformation of the actuators.^[^ ^27^ ^]^ Therefore, a programmable shape transformation can be realized by creating a well‐designed magnetization profile in SMRAs. Currently, examples of magnetization programming methods for SMRAs include but are not limited to template‐assisted methods,^[^ ^8^, ^28^ ^]^ printing ferromagnetic domains,^[^ ^16^, ^29^, ^30^ ^]^ light/heat‐aided magnetization,^[^ ^5^, ^31^, ^32^, ^33^ ^]^ and 4D printing‐assisted methods,^[^ ^19^, ^34^ ^]^ which have achieved various spatial and nonuniform distributions of magnetization profiles. However, it is challenging for the existing methods to encode complex direction‐varying magnetization profiles in 3D structures. For instance, the distribution of magnetic moments is relatively simple for discretized programming methods including printing ferromagnetic domains and light/heat‐aided magnetization methods. As a result, it remains a significant challenge to use existing methods to fabricate SMRAs with versatile morphing modes (e.g., out‐of‐plane buckling and cut‐guided deformation) and functionalities, specifically when the desired configuration and magnetization profiles are highly complex. New programming and fabrication methods are urgently required to expand the functions of soft magnetic machines further.

Kirigami, an ancient paper cutting art, has received significant attention as a promising strategy for 2D‐to‐3D shape transformation by paper cutting and cut‐guided out‐of‐plane deformation. The kirigami designs enable the initial basic structure to achieve incredible mechanical deformation ability,^[^ ^35^, ^36^, ^37^ ^]^ which has widespread applications across different areas, including flexible electronics,^[^ ^38^, ^39^ ^]^ soft actuators,^[^ ^40^, ^41^ ^]^ and metamaterials.^[^ ^42^, ^43^, ^44^ ^]^ Undoubtedly, applying the kirigami art to the fabrication and programming of SMRAs can realize a wide range of complicated 3D deformation modes. Therefore, research on the combination of kirigami design and SMRAs is urgently required to address these challenges.

Herein, we report a novel method for fabricating programmable SMRAs with numerous complicated shape morphing modes and bio‐inspired locomotion. The kirigami design strategy was applied to obtain 3D‐assembled configurations with out‐of‐plane buckling and cut‐guided deformation modes. The 3D‐assembled structures were magnetized by an impulse magnetic field to obtain 3D complicated and continuously varying magnetization profiles. This study first illustrates the entire process of fabricating SMRAs using the kirigami‐inspired method. Thereafter, the magnetic properties were characterized, and an integrated finite element analysis (FEA) method was developed to quantitatively analyze the shape morphing of the SMRAs. Various magnetoresponsive 3D curved morphologies with complex Gaussian curvatures are presented to illustrate the feasibility of this strategy for preparing SMRAs with novel morphing modes. Furthermore, three face‐like shapes with different expressions and a bio‐inspired scaled soft crawling robot were explored. The results indicate that the kirigami‐inspired method provides new ideas for the design and fabrication of SMRAs and broadens their application in diverse fields.

## 2 Results and Discussion

**Figure 1:**
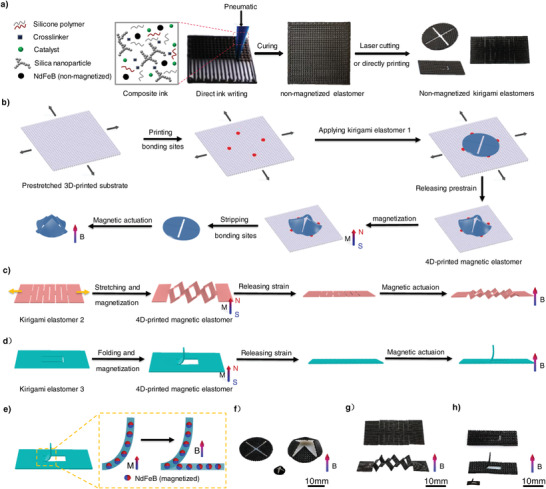
Schematic of kirigami‐inspired SMRAs fabricated via a DIW‐kirigami design‐mechanical assembly‐magnetization method. a) Schematic of the composition of composite magnetic ink, DIW, and kirigami design. b) Schematic of the key steps for buckling‐guided assembly method. c) Schematic of the key steps for stretching‐guided assembly method. d) Schematic of the key steps for folding‐guided assembly method. e) Schematic of the deformation mechanism of the SMRA under magnetic actuation. f–h) Experimental results of three kirigami‐inspired SMRAs rapidly transformed into complicated 3D structures under magnetic actuation. The actuation of the SMRA was performed with an applied magnetic field of approximately 150 mT generated by a permanent magnet. *M* in (b–h) represents the impulse magnetic field for magnetization and *B* represents the applied magnetic field for actuation. Dimensions of the 3D‐printed magnetoelastic kirigami sheets demonstrated in (f–h) are shown in Figure S4a–c (Supporting Information).

The procedures for fabricating the kirigami‐inspired programmable SMRAs include four main steps: direct ink writing (DIW), kirigami design, mechanical assembly, and magnetization. First, the magnetoelastic sheets were printed via DIW using composite magnetic ink. The magnetic ink was composed of a polydimethylsiloxane (PDMS) matrix embedded with non‐magnetized NdFeB particles. Silica nanoparticles were added to make the composite ink exhibit the flow behavior of the plastic fluid under external air pressure (Figure S1, Supporting Information). The printed magnetoelastic sheets were then cured in a vacuum‐drying oven. Second, the magnetoelastic sheets were cut using a carbon dioxide laser under the guidance of the kirigami design (**Figure** **1**a). Notably, DIW can enable us to print structures with kirigami design directly and fabricate complex 3D shapes. Laser cutting technology can be used to fabricate finer cuts on planar structures than DIW (Figure S2, Supporting Information). Moreover, laser cutting is a high‐throughput strategy because it can cut multiple kirigami samples simultaneously with high speeds (up to 50 mm s^−1^), as shown in Figure S3 (Supporting Information). Therefore, in this study, laser cutting was adopted to fabricate planar kirigami structures, and 3D structures with kirigami design were directly printed via DIW. Third, the magnetoelastic kirigami sheets were deformed into complicated 3D shapes using three typical mechanical‐guided assembly methods: buckling, stretching, and folding. Finally, the 3D‐assembled structures were magnetized to saturation under an impulse magnetic field (≈3 T). Consequently, 3D complicated and continuously varying magnetization profiles were obtained in the SMRAs after the internal stress during the assembly process was released. The SMRAs undergo shape morphing and evolve into a 3D structure similar to the 3D‐assembled configuration (Figure 1b–d) under magnetic actuation, which allows us to predict the responsive configuration before magnetization. The kirigami beam shown in Figure 1e was used as an example to explain the mechanism of the deformation behavior of SMRA under magnetic actuation. The impulse magnetic field causes the magnetic pole direction of all the NdFeB particles to align in the direction of the impulse field. After applying an actuation magnetic field, the hard particles induced torque and rotated until their magnetic domains were close to the direction of the applied magnetic field (Figure 1e). Hence, complex shape transformations can be realized by designing the distribution of the magnetization profiles.

In general, for the buckling‐guided assembly method, an elastomer substrate and a magnetoelastic sheet are first printed using the DIW method. The elastomer substrate was stretched to a certain strain using a homemade biaxial stretching device. Thereafter, four joints were printed on the substrate. The joints are located on the edges around the elastic sheet, as indicated by the red dots in Figure 1b. The elastomer substrate and magnetoelastic sheet are connected through these joints. After releasing the prestrain of the substrate, the magnetoelastic sheet evolves into a 3D curved structure with a complicated shape owing to cut‐guided deformation. For the stretching‐guided assembly method, a magnetoelastic sheet with periodic cuts was stretched, as shown in Figure 1c. The kirigami sheet morphs into a type of pop‐up 3D structure via out‐of‐plane buckling.^[^ ^45^ ^]^ Similarly, in the folding‐guided assembly method, the kirigami sheet was locally folded around the crease to obtain a 3D structure (Figure 1d). The experimental results of the three examples are presented to verify the ability of our methods to fabricate SMRAs (Figure 1f–h; Movie S1, Supporting Information). The magnetoelastic kirigami sheets can rapidly transform into complicated 3D structures under an applied magnetic field (≈150 mT), and the deformation processes are reversible. Moreover, the kirigami‐inspired method is suitable for fabricating millimeter‐centimeter‐scale SMRAs (Figure 1f,h). The results demonstrated that complex magnetization profiles and shape morphing can be obtained using the kirigami‐inspired method.

**Figure 2:**
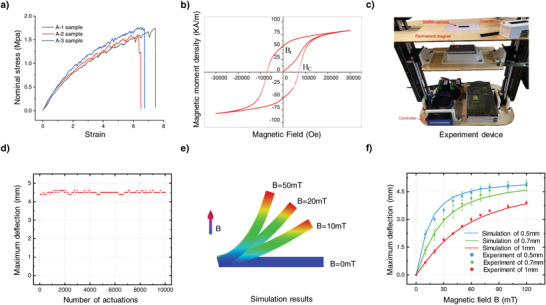
Properties of the SMRAs, and quantitative comparisons of the deformation given by FEA and experiments. a) Nominal tensile stress–strain curves of the magnetic soft materials. b) The hysteresis loop of the magnetic soft materials. c) Experiment device for testing the sustained driving performance of the SMRA. d) Relationship between the maximum deflection of the kirigami beam and the number of actuations. e) FEA of the deformation behavior of the kirigami beam (dimension: 5.2 × 3 × 0.5 mm) under magnetic actuation. f) Comparisons of FEA and experimental results of the maximum deflections of the kirigami beams with different dimensions (dimension: 5.2 × 3 × 0.5, 5.2 × 3 × 0.7, and 5.2 × 3 × 1 mm) under applied magnetic fields. The content of hard magnetic particles in magnetic soft materials is 38.4 wt.%.

In contrast to the previous rigid kirigami paper, owing to its low elastic modulus and high break elongation (>600%, **Figure** **2**a), the 3D‐printed magnetoelastic sheet exhibits nonlinear deformation behavior during mechanical‐guided assembly and magnetic actuation. To quantitatively analyze the deformation mechanisms of the kirigami‐inspired SMRAs, the constitutive model of ideal hard magnetic soft materials established by Zhao et al.^[^ ^46^ ^]^ based on the neo‐Hookean continuum model is adopted here, which gives the Cauchy stress tensor as $\sigma \;=\;\mu J^{-\frac{5}{3}}\left(FF^{T}-\frac{I_{1}}{3}I\right)+K\left(J-1\right)I-J^{-1}B⊗M$. Here, *μ*, *J*, *F,B*, and *M* denote the shear modulus, volumetric Jacobian, deformation gradient tensor, actuation magnetic field, and magnetization vector, respectively. *K* is the bulk modulus and *I*_1_ = *tr*(**F** ^T^ **F**). The magnetization profiles in the SMRAs were calculated according to **M** = **F** ^−1^ **M**_0_ (Figure S5, Supporting Information), where $M_{0}=\;m{\left[\begin{array}{ccc} 0 & 0 & 1 \end{array}\right]}^{T}$ is the magnetization profile in the 3D‐assembled configuration. The direction of *M*_0_ is consistent with that of the impulse magnetic field. From the Cauchy stress tensor, it can be concluded that the magnetic moment contribution disappears when the magnetization vector is aligned with the actuating magnetic field. Hence, the magnetic moment causes the soft actuator to rotate until the magnetization profile aligns with the direction of the actuating magnetic field (Figure S6, Supporting Information). The remanent magnetization of the magnetoresponsive material can be obtained from its hysteresis loop, which demonstrates that the influence of the actuating field on the remanent magnetization of the SMRA can be ignored (Figure 2b; Table S1, Supporting Information). The maximum deflections of the kirigami beam under 10 000 cycles of actuation were tested to characterize the sustained driving performance of the SMRA (Figure 2c,d; Movie S2, Supporting Information). The results indicated that the fabricated SMRA exhibited good robustness.

The large deformation behavior of SMRAs can be quantitatively predicted using an integrated FEA method based on the above theoretical framework. The details of the FEA method are provided in the Experimental Section. The kirigami beam in Figure 1e is considered as the basic structure to quantitatively analyze the accuracy of the FEA method and the key factors influencing the actuation performance of the SMRA. When the kirigami beam is placed within a uniform magnetic field, as shown in Figure 1e, the maximum deflection of the SMRA can be scaled as^[^ ^16^, ^46^ ^]^ $\;\delta_{max}\propto \frac{MBL^{3}}{\mu H^{2}}$, where *L* and *H* are the length and height of the kirigami beam, respectively. The equation qualitatively reveals that the deflection of SMRA can be tuned by regulating the actuating field, magnetization, shear modulus, and length‐to‐thickness ratio. To validate the capability of the FEA method to quantitatively predict the deformation behavior of SMRAs, comparisons of FEA and experimental results for the maximum deflections of kirigami beams with different dimensions under magnetic actuations were conducted (Figure 2e,f), which are consistent. The results demonstrate that it is feasible to predict the deformation behavior of SMRAs using the integrated FEA method.

**Figure 3:**
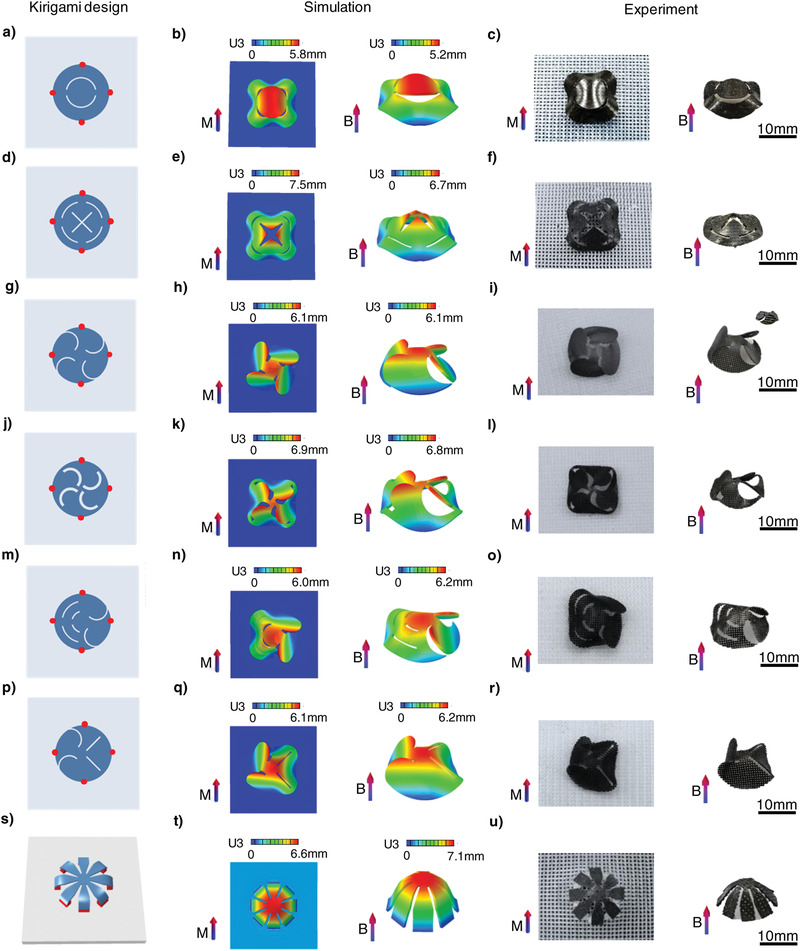
Kirigami‐inspired SMRAs fabricated via compression‐induced buckling‐guided magnetization. a–u) Kirigami designs, simulations, and experimental results of the deformed samples under compression‐induced buckling and applied magnetic fields of ≈150 mT for 3D surfaces with a–f) symmetric cut patterns, g–l) antisymmetric cut patterns, m–r) asymmetric cut patterns, and s–u) a 3D sphere shape. Equibiaxial pre‐strain in substrate is 40% in (a–r) and 60% in (s–u). *M* in (a–u) represents the impulse magnetic field for magnetization and *B* represents the applied magnetic field for actuation. Dimensions of the kirigami designs demonstrated in (a–u) are shown in Figure S4d–j (Supporting Information).

Various cut patterns on basic circular sheets are presented to illustrate the versatility of our method for preparing SMRAs with complicated 3D shapes (**Figure** **3**). According to the characteristics of the cut patterns on the magnetoelastic sheets, the kirigami design can be divided into three categories: i) symmetric, ii) antisymmetric, and iii) asymmetric cut patterns. Magnetization programming of circular sheets with kirigami designs was performed through compression‐induced buckling. The SMRAs can rapidly deform into complicated 3D curved structures under an applied magnetic field of ≈150 mT. Figure 3a,d show a schematic of circular kirigami sheets with symmetric cuts, whose 3D‐assembled shapes and responsive configurations under magnetic actuation are 3D surfaces with symmetric Gaussian curvature (Figure 3a–f; Movie S3, Supporting Information). Similarly, arc‐shaped cuts provide antisymmetric kirigami design examples, which rapidly transform into 3D curved structures with antisymmetric Gaussian curvature under applied magnetic fields (Figure 3g–l; Movie S3, Supporting Information). Furthermore, two SMRAs with asymmetric kirigami designs were fabricated by adjusting the position and type of the cut patterns to obtain more complicated shape transformations (Figure 3m,p). They can evolve into 3D surfaces with complicated Gaussian curvatures via compression‐induced buckling and magnetic actuation (Figure 3m–r; Movie S3, Supporting Information). As a type of nondevelopable surface, the sphere cannot be acquired by directly compressing the circular sheets. Therefore, a set of symmetric cut patterns was designed. 3D‐printed wedge blocks were introduced as bonding sites to rotate the ends of the magnetoelastic sheet relative to the substrate (Figure S7, Supporting Information). As a result, a 3D sphere was obtained under an actuating magnetic field (Figure 3s–u; Movie S3, Supporting Information). The above experimental and FEA results confirm the feasibility of our strategy for obtaining 3D cured shapes with complicated Gaussian curvatures. Moreover, the curvature of the SMRAs under magnetic actuation can be programmed by the design of the kirigami patterns.

**Figure 4:**
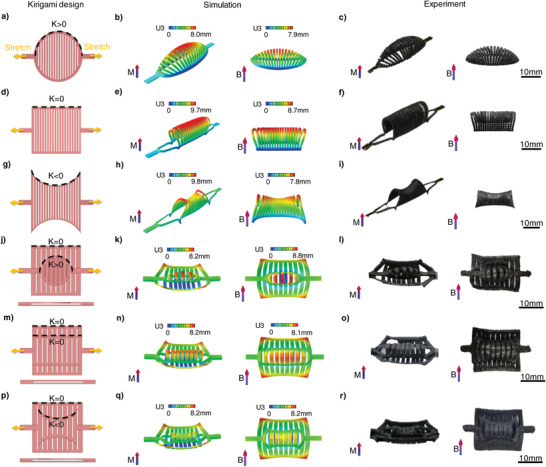
Kirigami‐inspired SMRAs fabricated via stretching‐guided magnetization. a–r) Kirigami designs, simulations, and experimental results of deformed samples under stretching‐induced deformation and applied magnetic fields of ≈150 mT for a–c) an ellipsoid‐shaped structure; d–f) a cylindrical structure; g–i) a saddle structure; j–l) a multilayer structure with “zero + positive” Gaussian curvatures; m–o) a multilayer structure with “zero + zero” Gaussian curvatures; p–r) a multilayer structure with “zero + negative” Gaussian curvatures. The tensile displacements of both ends of the kirigami sheet are 4 mm in (a–c), 9 mm in (d–f), 12 mm in (g–i), and 4 mm in (j–r). *M* in (a–r) represents the impulse magnetic field for magnetization and *B* represents the applied magnetic field for actuation. Dimensions of the kirigami designs demonstrated in (a–r) are shown in Figure S4k–p (Supporting Information).

Previous research have shown that 2D‐to‐3D shape morphing of kirigami sheets based on out‐of‐plane buckling or rigid rotation can be obtained through stretching‐induced stress.^[^ ^43^ ^]^ Recently, 3D surfaces with a global Gaussian curvature were obtained by stretching kirigami sheets with a programmed boundary curvature.^[^ ^35^ ^]^ Inspired by this mechanism, a magnetoelastic circular sheet with a positive boundary curvature and a set of parallel cuts was prepared (**Figure** **4**a). It transforms into an ellipsoidal 3D structure when both ends of the kirigami sheet are stretched. The structure was then magnetized under an impulse magnetic field. After tensile strain was released, the structure reverted to its original shape. Finally, the SMRA rapidly transformed into a similar ellipsoidal structure with a positive Gaussian curvature under an applied magnetic field (Figure 4b,c; Movie S4, Supporting Information). Similarly, a cylindrical 3D structure with zero Gaussian curvature was obtained under magnetic actuation when the upper and lower boundaries of the 3D‐printed magnetoelastic sheet were cut into straight lines (Figure 4d–f; Movie S4, Supporting Information). Moreover, the SMRA transformed into a saddle 3D surface with negative Gaussian curvature under an applied magnetic field if its boundaries were cut into curves with negative curvature (Figure 4g–i; Movie S4, Supporting Information). The DIW technique enables us to perform kirigami design on 3D complex multilayer structures, which cannot be fabricated by conventional methods such as compression molding. Subsequently, a few 3D multilayer structures with well‐designed cut patterns were printed using a supporting ink (Figure 4j–r). First, a multilayered kirigami structure with “zero + positive” boundary curvatures was designed and printed. Each elastic ribbon of the kirigami structure pops up or down via stretching and cut‐guided deformation. After the multilayered kirigami structure was programmed by stretching‐guided magnetization, it transformed into an elliptical surface and a cylindrical surface with “zero + positive” Gaussian curvatures (Figure 4j–l; Movie S4, Supporting Information). Similarly, two multilayered kirigami structures with “zero + zero” and “zero + negative” boundary curvatures were designed, printed, and programmed using this method. Consquently, they deformed into complex 3D curved morphologies with “zero + zero” and “zero + negative” Gaussian curvatures (Figure 4m–r; Movie S4, Supporting Information). Therefore, the 3D curved morphologies of SMRAs with programmed Gaussian curvatures under magnetic actuation can be designed and fabricated by manipulating the boundary curvatures and the kirigami design.

**Figure 5:**
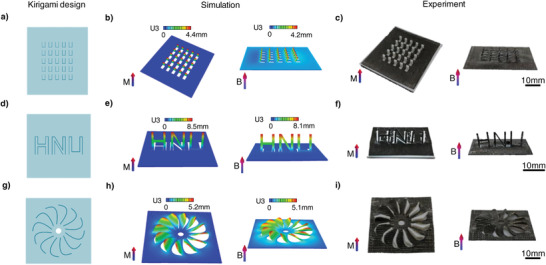
Kirigami‐inspired SMRAs fabricated via folding‐guided magnetization. a–i) Kirigami designs, simulations, and experimental results of deformed samples under folding‐induced deformation and applied magnetic fields of ≈150 mT for a–c) an array structure, d–f) a 3D structure with “HNU” pattern, and g–i) a 12‐blade propeller structure. *M* in (a–i) represents the impulse magnetic field for magnetization and *B* represents the applied magnetic field for actuation. Dimensions of the kirigami designs demonstrated in (a–i) are shown in Figure S4q–s (Supporting Information).

Previous scholars have prepared SMRAs with 3D shape morphing using an origami‐inspired magnetic programming strategy.^[^ ^20^, ^47^ ^]^ Taking advantage of this method, various cut patterns were designed on a magnetoelastic sheet. All the cut patterns retained an edge as a crease to connect with the matrix material. Therefore, the kirigami sheets can be transformed into 3D structures under magnetic actuation through folding‐guided deformation and magnetization programming. For instance, a 5 × 5 array pattern was cut on a magnetoelastic sheet (**Figure** **5**a). Subsequently, each unit of the array pattern was vertically folded and then magnetized under an impulse magnetic field. The magnetoelastic sheet reverts to its original shape after the stress is released. As a result, the magnetoelastic sheet rapidly transformed into a 3D cilia‐shaped structure under magnetic actuation (Figure 5b,c; Movie S5, Supporting Information). Moreover, an “HNU” pattern was cut on the magnetoelastic sheet, and magnetization programming was conducted using the folding‐guided method. The 2D pattern of SMRA bends close to 90° under an external magnetic field (Figure 5d–f; Movie S5, Supporting Information). A set of arc‐shaped cut patterns with a central antisymmetric distribution on the magnetoelastic sheet was also designed (Figure 5g). Each unit of the kirigami sheet was folded along the crease and magnetized to obtain a SMRA. The SMRA could transform into a 12‐blade propeller structure under magnetic actuation (Figure 5h,i; Movie S5, Supporting Information). The SMRAs obtained by the kirigami‐inspired method may find wide applications in the fields of color‐switchable devices,^[^ ^48^ ^]^ metamaterials, and bionic robotics.^[^ ^49^ ^]^

**Figure 6:**
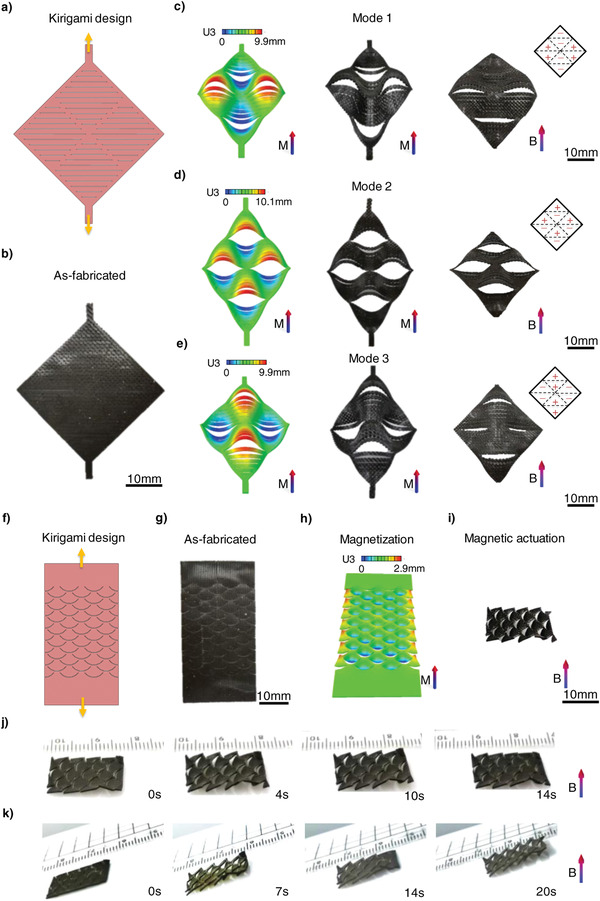
Functional demonstrations of the kirigami‐inspired SMRAs. a,b) Kirigami design and as‐fabricated sample of a diamond‐shaped sheet. c–e) Simulation results of the deformed samples under stretching‐induced deformation and corresponding experimental results through stretching and magnetic actuating for 3D face‐like shape with c) smiley, d) exciting, and e) sad expressions. The tensile displacement of both ends of the kirigami sheet is 4 mm in (c–e). f,g) Kirigami design and the as‐fabricated sample of a rectangle sheet. h,i) Simulation result of a scaled shape structure under stretching‐induced deformation and corresponding experimental result through magnetic actuating. j) A kirigami‐inspired scaled soft crawling actuator. k) The scaled soft crawling actuator climbs a ramp. *M* in (a–k) represents the impulse magnetic field for magnetization and *B* represents the applied magnetic field for actuation. Dimensions of the kirigami designs demonstrated in (a,f) are shown in Figure S4t,u (Supporting Information).

Face‐changing is a traditional art in ancient China that reflects the inner emotions of characters by changing their face morphologies. Inspired by this ancient art, a diamond‐shaped kirigami sheet composed of 2 × 2 units with a set of parallel cuts was designed and fabricated (**Figure** **6**a,b). Each elastic ribbon of the kirigami sheet pops up or down via stretching and cut‐guided deformation. Therefore, each unit had a typical multistable structure. Furthermore, the diamond‐shaped kirigami sheet can be divided into eight independent pop‐up areas according to human facial features. The face morphology can be regulated by controlling the steady state of each pop‐up area (Figure 6c‐e). “+” and “‐” represent pop up and pop down in the inset of Figure 6c–e, respectively. A smiling face shape was obtained when the top and bottom units popped down and the units on both sides popped up. The 3D face‐like structure was magnetized to saturation under an impulse magnetic field perpendicular to the kirigami sheet. The structure reverted to its original diamond shape after the tensile constraint was released. Finally, the kirigami sheet was transformed into the smiling face mode under an applied magnetic field (Figure 6c; Movie S6, Supporting Information). Likewise, a face‐like SMRA with an exciting expression was obtained when elastic ribbons on the top half of each unit popped up, and the rest popped down (Figure 6d; Movie S6, Supporting Information). Furthermore, SMRA with a sad expression can be obtained when the structure is magnetized in the 3D‐assembled configuration with the units on two sides popping down and other units popping up (Figure 6e; Movie S6, Supporting Information). Hence, face morphologies with different expressions could be programmed by controlling the steady state of each kirigami ribbon.

Many animals exploit the special friction properties of their skin to propel themselves. For instance, snakes rely on the frictional anisotropy of their scaled skin for movement.^[^ ^41^, ^50^, ^51^ ^]^ Inspired by the friction‐assisted locomotion mechanism, a soft kirigami sheet that mimics the shape and arrangement of snakes' skin was designed and fabricated using our method (Figure 6f,g). The stretchable kirigami sheet transformed into a 3D‐textured surface similar to the skin of snakes through uniaxial stretching and cut‐guided deformation (Figure 6h). The 3D kirigami structure was then magnetized to saturation under an impulse magnetic field. The perimeter of the kirigami sheet was cut to prevent edge buckling induced by stretching and magnetic actuation (Figures S4u and S8, Supporting Information). Consequently, the SMRA transforms into a 3D structure akin to the scaled skin of snakes under a uniform magnetic field (Figure 6i; Movie S7, Supporting Information). Meanwhile, this type of highly directional 3D feathers makes the SMRA obtain anisotropic friction. Hence, the fabricated SMRA could continuously crawl forward by repeatedly applying and removing the magnetic field generated by a permanent magnet, which is similar to the movements of snakes (Figure 6j; Movie S7, Supporting Information). More importantly, the SMRA has the ability to crawl on sloped terrain with an angle of ≈20° (Figure 6k; Movie S7, Supporting Information). This type of bionic soft crawling robot may have various innovative applications, such as environmental exploration and clinical treatments.

## 3 Conclusion

In summary, we propose a novel strategy for utilizing the kirigami‐inspired method to guide the fabrication and programming of SMRAs with versatile morphing modes, including out‐of‐plane buckling and cut‐guided deformation. This method was validated using various samples based on theoretical analysis, FEA, and experiments. Compared with existing methods, such as the template‐assisted method, printing ferromagnetic domains, and light/heat‐aided methods, the proposed methods can easily realize programmable magnetoresponsive 3D curved shapes of SMRAs with complicated Gaussian curvatures. Complex SMRAs can be further explored by designing the cut patterns, boundary curvatures, and dimensions of the kirigami structures. It is highly desirable to create such SMRAs because they can obtain a more diverse range of shape‐morphing modes, locomotive gaits, and functions. However, some limitations should be addressed for further development of the kirigami‐inspired SMRA. The main limitation is that the sizes of the SMRAs are limited by our manufacturing technology, which makes it difficult to fabricate SMRAs below the millimeter scale. Utilizing other fabrication techniques with higher resolution,^[^ ^52^ ^]^ kirigami‐inspired miniature SMRAs can be obtained. The proposed methods can also be applied to prepare multifunctional SMRAs using other material systems such as shape memory polymers^[^ ^53^ ^]^ and reconfigurable materials.^[^ ^31^, ^33^ ^]^ This study provides a novel way to create SMRAs with versatile morphing modes and it may find potential applications in the fields of soft machines, color‐switchable devices, soft grippers, and flexible electronics.

## 4 Experimental Section

### Composite Ink Preparation

For the ink system of the magnetic sheets, a polymer matrix was prepared by mixing two common PDMS materials: SE 1700 base (Dow Corning Corp.) and Ecoflex‐10 Part B (Smooth‐on Inc.) in a mass ratio of 1:1. Fumed SiO_2_ nanoparticles (2.5 wt.% with respect to the matrix) were added to the polymer matrix to obtain shear thinning and shear yield properties. Thereafter, NdFeB microparticles (66.67 wt.% with respect to the polymer matrix, LW‐BA 16–7A, Xinnuode Corp.) with an average size of 5 µm and the SE 1700 catalyst (10 wt.% with respect to SE 1700 base) were added into the prepolymer. The mixture was then blended thoroughly at 1500 rpm for 4 min using a planetary mixer (ITT‐300S, Integrity) to obtain a homogeneous blend. Finally, the composite ink was centrifuged at 4000 rpm for 3 min to remove gas bubbles before 3D printing. The ink system of the substrates was prepared by mixing and centrifuging the SE 1700 base and the catalyst in a 10:1 weight ratio. The polypropylene oxide (CELLINK START, Cellink Company.) was used as the supporting ink for printing the 3D multilayer structures. After the magnetic ink was fully cured in a vacuum‐drying oven at 100 °C for 5 h, the supporting ink was removed by dissolving it in water.

### Kirigami Magnetoelastic Sheet Preparation

First, magnetoelastic sheets were printed using a 3D bio‐printer (BioX, Cellink Company) using the prepared composite magnetic ink. The composite ink was extruded through a nozzle (diameter, 410 or 250 µm) with controlled gas pressure (200–400 kPa) and printing speed (10–40 mm s^−1^) according to the viscosity of the ink and the dimensions of the printed structures. The printed magnetoelastic sheets were then solidified in a vacuum drying oven at 100 °C for 5 h. The 3D‐printed magnetoelastic sheets were then cut using a commercial CO_2_ laser cutting machine (E1309M, ZhengTianHengye CNC Corp.). The power and speed of the laser were 10 W and 30 mm s^−1^, respectively. Finally, the kirigami magnetoelastic materials were magnetized under an impulse field (≈3 T) generated by a commercial magnetizer (PFD‐2000, Tianjie Magnetoelectric Technology Corp.) after mechanically guided assembly.

### Mechanical Characterization

The uniaxial tensile tests were performed on a universal tensile testing machine (ZQ‐990, Zhiqu precision instruments corp.) at a displacement rate of 30 mm min^−1^. The dimensions of the magnetoelastic and substrate samples were 40 × 10 × 0.8 mm and 50 × 10 × 2 mm, respectively. Nominal stress–strain curves were plotted for all the samples. The shear moduli of the magnetoelastic materials and substrates were obtained by fitting the experimental curves to the neo‐Hookean model, which were 230 and 290 kPa, respectively (Figure 2a; Figure S9, Supporting Information).

### Magnetic Characterization

The magnetic moments of the magnetic materials were characterized using a physical property measurement system (Dynacool 9, Quantum Design). The samples were prepared by 3D printing of a cuboid structure (dimensions:10 × 10 × 4 mm) and then cut into a cylinder using a 3‐mm biopsy punch (Miltex Inc.). All experiments were performed at 25 °C. The remanent magnetization of the specimens was calculated by dividing the residual magnetic moment by volume. The volume was calculated based on the mass and density of the sample.

### FEA

The simulation method consisted of three steps. First, FEA was performed using the software ABAQUS (2016) to obtain the 3D‐assembled shape, whose deformation gradient tensor was output. The magnetic sheet and substrate were considered solid bodies because the dimensions of the structures were significantly larger than the lattice spaces. The kirigami sheets were tied to the substrates through the joints. To account for the nonlinear and large deformation behavior of the material, the materials were modeled as nearly incompressible neo‐Hookean materials. A first‐order hybrid element (C3D8RH) was used. The thermal expansion coefficient and cooling load were used to simulate the release of the prestretched substrate. Initial geometric imperfections were introduced into the model to better trigger the deformation behavior of the structure. Second, magnetization profile *M* can be calculated based on the deformation gradient tensor using a custom program in MATLAB. Finally, the shape evolution of the SMRA under an applied magnetic field can be obtained based on the magnetization profile and a user‐defined element subroutine implemented in ABAQUS. The input parameters were as follows: shear modulus *μ*  = 230 kPa for the magnetic material and *μ* = 290 kPa for the substrate material, bulk modulus *K* = 100G, magnetizations of magnetic material *M*_r_ = 50 kA m^−1^, and applied magnetic field *B* = 150 mT.

## Conflict of Interest

The authors declare no conflict of interest.

## Supporting information

Supporting Information

Supplemental Movie 1

Supplemental Movie 2

Supplemental Movie 3

Supplemental Movie 4

Supplemental Movie 5

Supplemental Movie 6

Supplemental Movie 7

## Data Availability

The data that support the findings of this study are available from the corresponding author upon reasonable request.
