# Supplementary material for: Kirigami‐Inspired Programmable Soft Magnetoresponsive Actuators with Versatile Morphing Modes
Source: Adv Sci (Weinh). 2022 Sep 30;9(32):2203711. doi: 10.1002/advs.202203711 (PMC9661843; doi:10.1002/advs.202203711)
Supplement: Supplementary file 1 — Supporting Information [file ADVS-9-2203711-s004.pdf]

Supporting Information

Kirigami-inspired programmable soft magnetoresponsive actuators with versatile morphing modes

Hanlin Zhu[#], Yuan Wang[#], Yangwen Ge, Yan Zhao, Chao Jiang**

H. Zhu, Y. Wang, Y. Ge, Prof. Y. Zhao, Prof C. Jiang

State Key Laboratory of Advanced Design and Manufacturing for Vehicle Body

College of Mechanical and Vehicle Engineering

Hunan University

Changsha 410082, P. R. China

Email: yanzhao@hnu.edu.cn (Y.Z.), jiangc@hnu.edu.cn (C.J.)

[#]These authors contributed equally to this work.

1. Supplementary Figures S1-S9

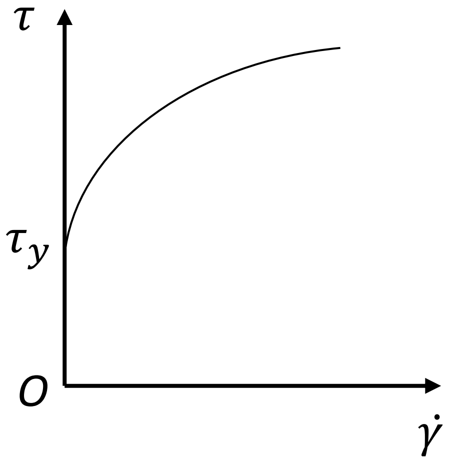

Figure S1. Flow diagram of plastic fluid. The non-Newtonian fluid have the shear thinning behavior and shear yield behavior as the apparent viscosity (slope) decreased along with the increased applied shear rate and the shear yield stress exists. These flow behaviors ensure that the composite ink can be smoothly squeezed out of a nozzle through an external pressure and maintain its shape during the printing process [1].

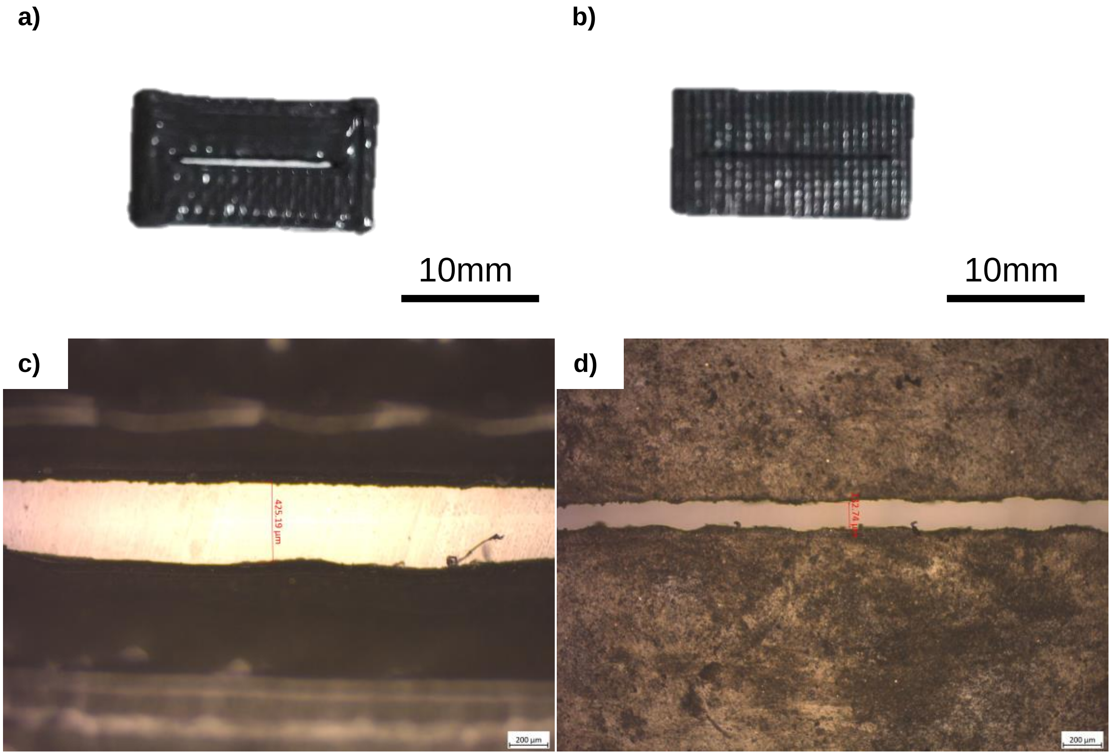

Figure S2. Optical microscope images of cut lines fabricated by direct ink writing and laser cutting. a, c) Cut pattern obtained by directly printing. b, d) Cut pattern fabricated by the laser cutting method.

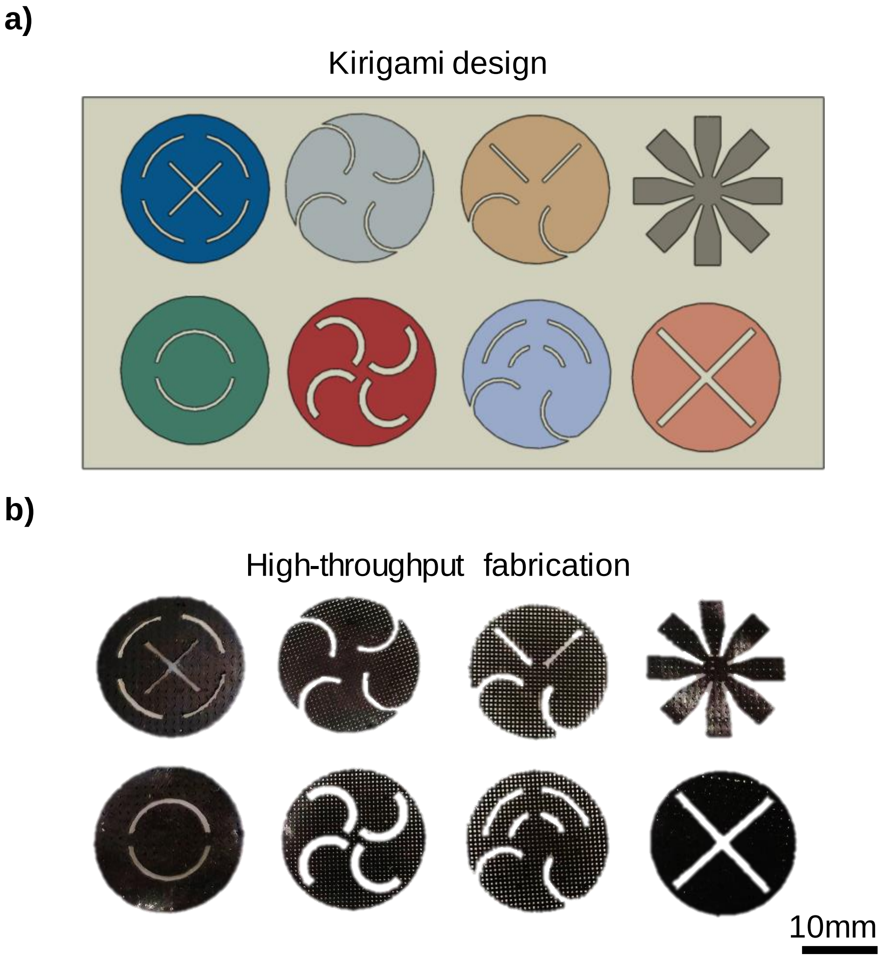

Figure S3. Kirigami design and high-throughput fabrication of eight samples on a planar sheet at one time.

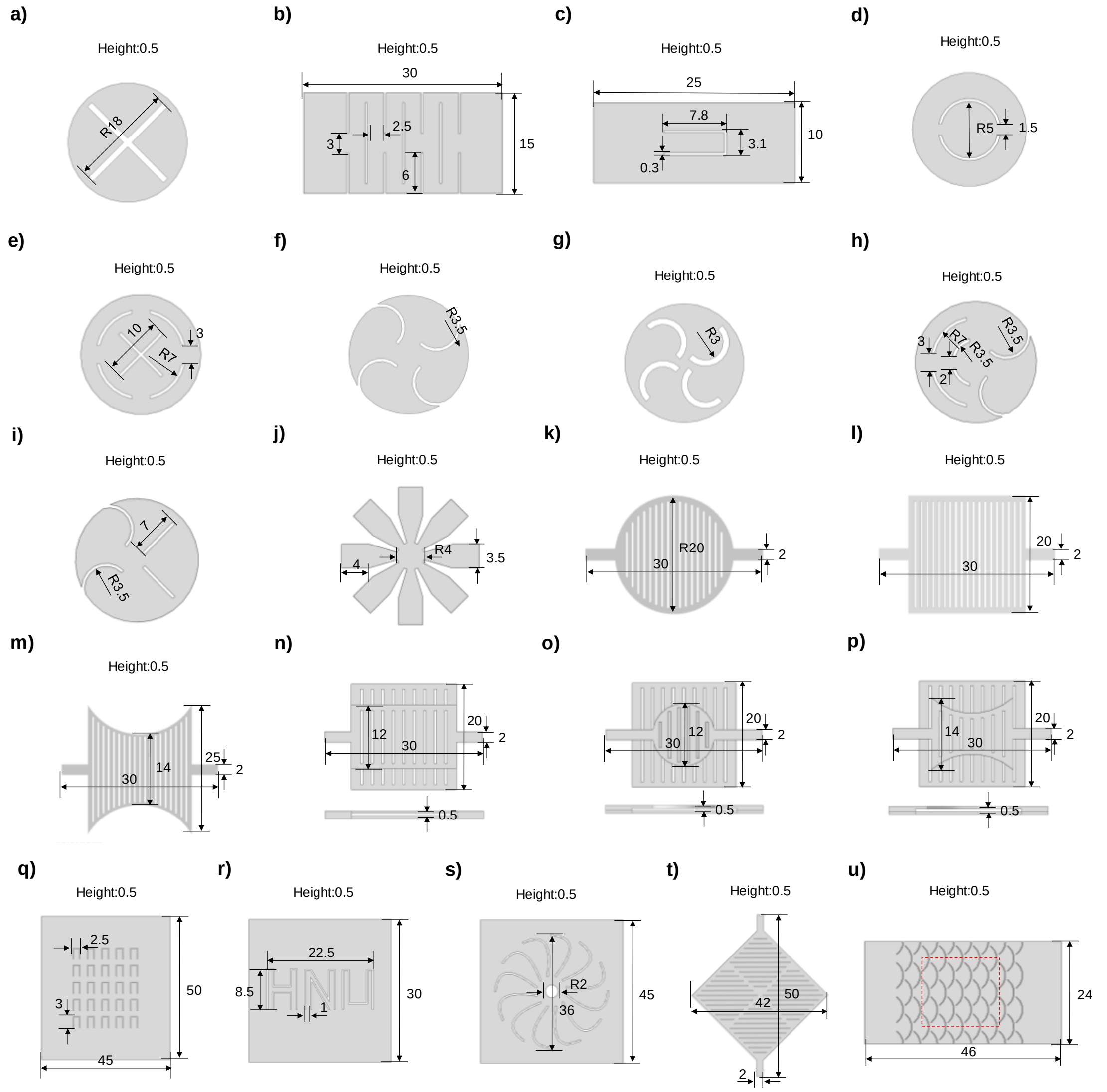

Figure S4. Dimensions of all kirigami samples demonstrated in Figure 1-6. a-c) Example of three kirigami precursors for buckling, stretching and folding methods. d, e) Circular sheets with symmetric cuts. f, g) Circular sheets with antisymmetric cuts. h, i) Circular sheets with asymmetric cuts. j) Precursor of kirigami sheets for the spherical structure. k-m) Precursors of kirigami sheets patterned with parallel cuts and different boundary curvatures. n-p) Precursors of multilayer structures patterned with parallel cuts and different boundary curvatures. q) Precursor for array structure. r) Square sheets with ‘HNU’ cuts. s) Precursor for 12-blade propeller. t) Precursors of kirigami sheets composed of 2 × 2 square units. u) Precursor for soft crawling robot.

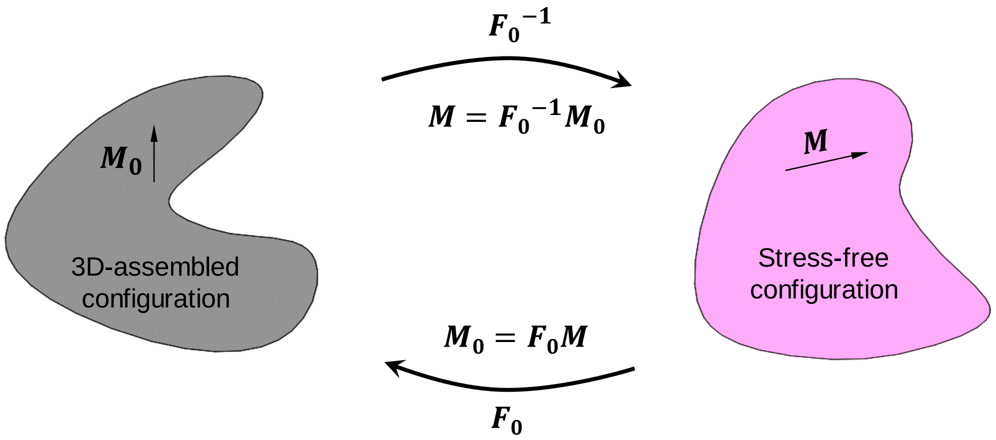

Figure S5. Schematic diagram of the relationship between the magnetization vectors in the stress-free and 3D-assembled configuration.

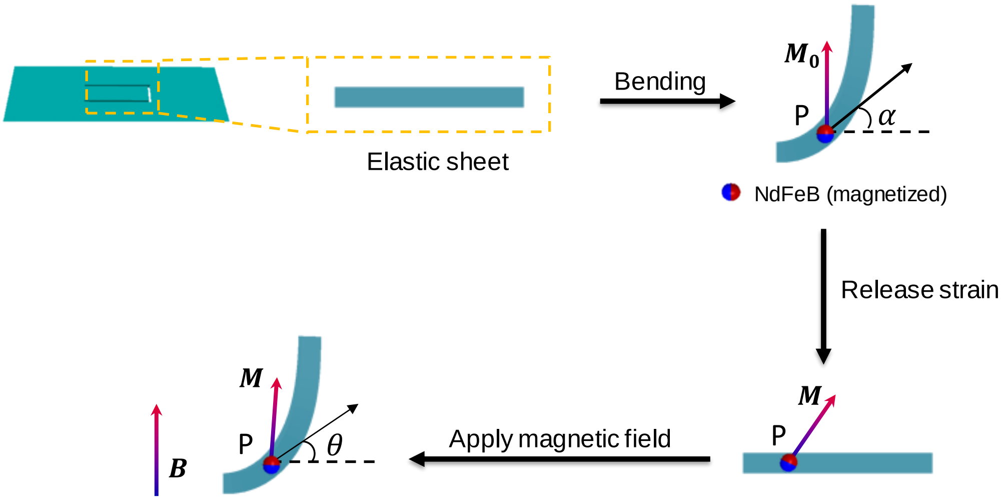

Figure S6. Schematic diagram of Kirigami-inspired SMRAs produced by DIW-kirigami design-mechanical assembly-magnetization method.

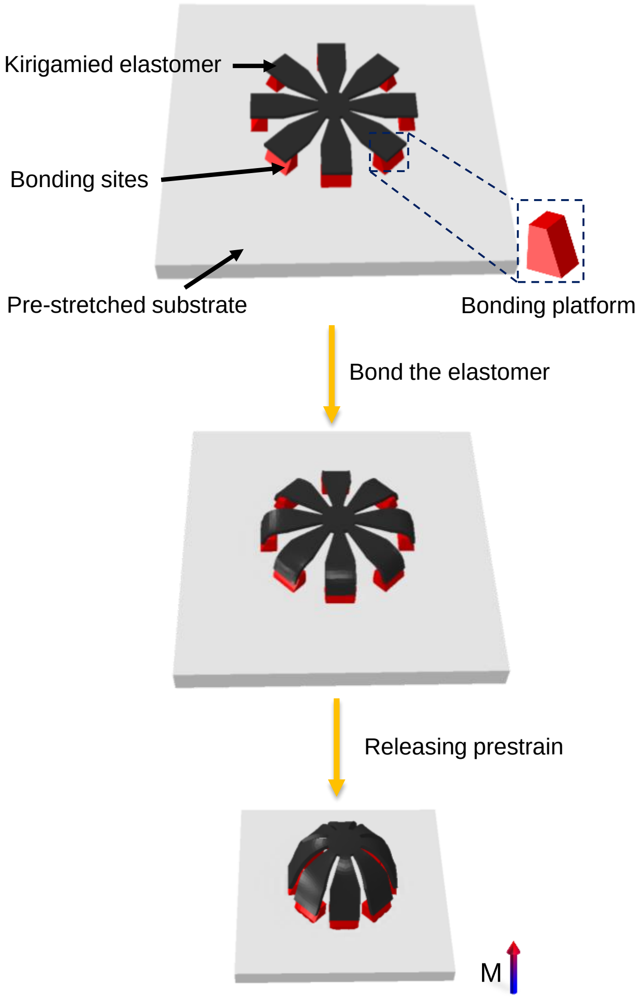

Figure S7. Schematic diagram of the steps to obtain the spherical structure using the kirigami-inspired method.

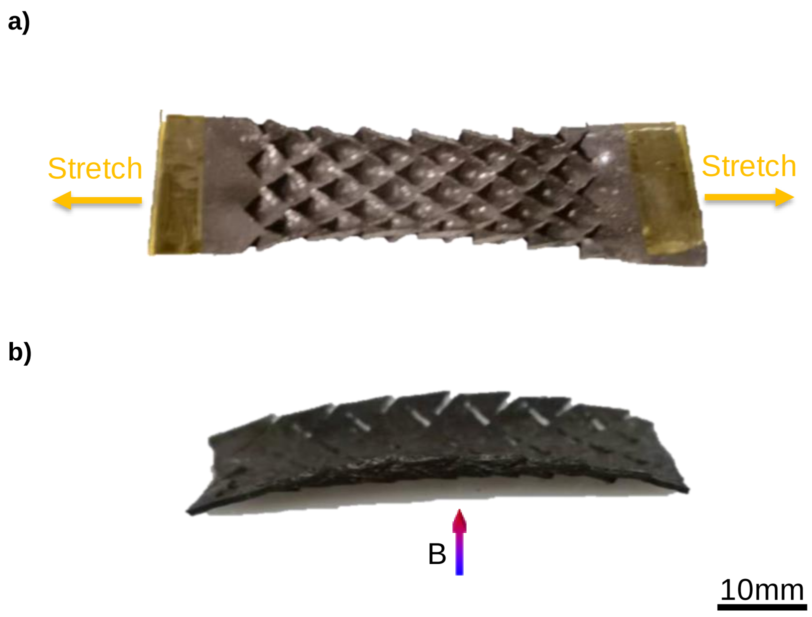

Figure S8. Experiment results of the kirigami sheet akin to the skin of snakes induced by stretching.

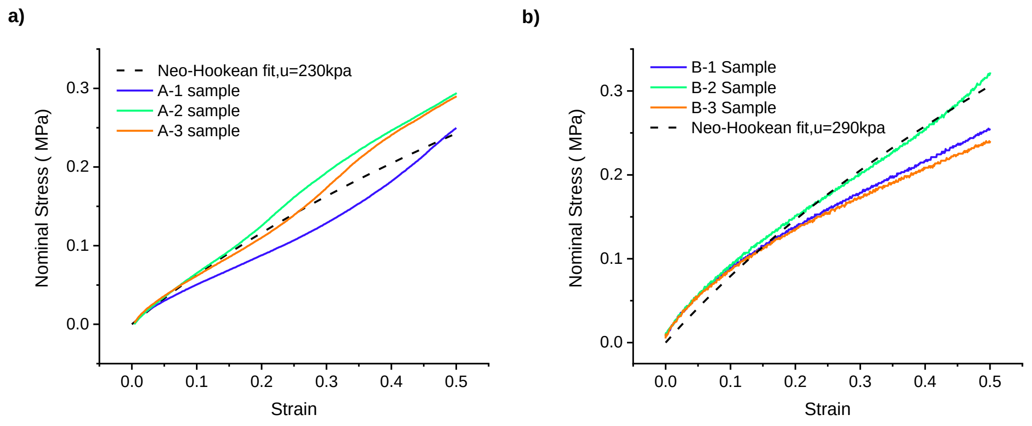

Figure S9. Mechanical characterizations of the 3D-printed materials. a, b) Nominal tensile stress–strain curves (solid lines) of the magnetized soft materials with magnetic particle content being 38.4wt% (a) and the substrate materials (b). The fitted curves (dashed lines) and corresponding parameters (shear modulus μ of the material) were obtained by fitting the experimental data to the nonlinear neo-Hookean model. The strain energy density function of the neo-Hookean model is given by $W = \frac{\mu}{2}(I_1 - 3) + \frac{K}{2}(J-1)^2$. Here μ and K donate the shear modulus and bulk modulus. J donate the volumetric Jacobian and $J = 1$ for incompressible material. I_1 is the first invariant of the Cauchy-Green deformed tensor, which is given by $I_1 = \lambda_1^2 + \lambda_2^2 + \lambda_3^2$. λ_i donate the principal stretches and $\lambda_1\lambda_2\lambda_3 = J$. For incompressible material subjected to uniaxial tension, $\lambda_2 = \lambda_3 = \sqrt{\frac{1}{\lambda_1}}$. Therefore, the strain energy density function can be simplified as $W = \frac{\mu}{2}\left(\lambda_1^2 + \frac{2}{\lambda_1} - 3\right)$. The constitutive model of neo-Hookean can be given by $\sigma = \frac{\partial W}{\partial \lambda_1} = \mu\left[1 + \varepsilon - \frac{1}{(1+\varepsilon)^2}\right]$. Here σ is the nominal stress and $\varepsilon = \lambda_1 - 1$ is the nominal strain. There is only one parameter (shear modulus μ) in the constitutive equation. Then, the shear modulus μ of the material can be obtained by fitting the experimental data to the above constitutive equation.

2. Supplementary Table S1

Table S1. Magnetic characterizations of the printed samples with composite magnetic inks.

Magnetic particle mass fraction [%]	Number of samples	Mass [g]	Volume [mm^3]	Magnetic moment [emu]	Magnetization [KA/m]
38.3	1	0.0358	12.99	0.669	51.50
	2	0.0336	12.20	0.648	53.11

The concentrations of the ink system were as follows: 28.7 wt% SE 1700 base, 28.7 wt% Ecoflex-10 Part B, 1.4 wt% fumed silica nanoparticles, 38.3 wt% NdFeB particles and 2.9 wt% SE 1700 catalyst. The average density of the samples measured by Archimedes' law is 2.755 g/cm3.

3. Supplementary Movies S1-S7

Movies S1.

Shape-morphing of three examples of SMRAs.

Movies S2.

Actuation experiment of SMRAs.

Movies S3.

Shape-morphing of kirigami-inspired SMRAs via buckling.

Movies S4.

Shape-morphing of kirigami-inspired SMRAs via stretching.

Movies S5.

Shape-morphing of kirigami-inspired SMRAs via folding.

Movies S6.

3D face shape-morphing SMRAs.

Movies S7.

Bionic scaled soft crawling robot.

Supplementary References

[1] H. Zhu, Y. He, Y. Wang, Y. Zhao, C. Jiang, *Adv. Intell. Syst.* **2021**, *4*, 2100137.
